# Case report: rare skeletal manifestations in a child with primary hyperparathyroidism

**DOI:** 10.1186/s12902-017-0197-z

**Published:** 2017-07-21

**Authors:** Maulee Hiromi Arambewela, Kamani Danushka Liyanarachchi, Noel P. Somasundaram, Aruna S. Pallewatte, Gamini L. Punchihewa

**Affiliations:** 1 Department of diabetes and endocrinology, National Hospital of Sri Lanka, Colombo, Sri Lanka; 2 Department of Radiology, National Hospital Sri Lanka, Colombo, Sri Lanka; 3 Orthopaedic Unit, National Hospital Sri Lanka, Colombo, Sri Lanka

**Keywords:** Primary hyperparathyroidism, Pubertal growth spurt, Genu valgum, Slipped upper femoral epiphysis, Epiphyseal displacement of humerus, Case report

## Abstract

**Background:**

Primary hyperparathyroidism (PHPT) is uncommon among children with an incidence of 1:300,000. This diagnosis is often missed in children in contrast to adults where it is detected at a pre symptomatic stage due to routine blood investigations. Etiology of PHPT can be due to adenoma, hyperplasia or rarely carcinoma.

**Case presentation:**

A 12 year old Sri Lankan girl presented with progressive difficulty in walking since 1 year. On examination she had bilateral genu valgum. Skeletal survey revealed valgus deformity of knee joints, bilateral subluxation of upper femoral epiphysis(SUFE), epiphyseal displacement of bilateral humeri, rugger jersey spine and subperiosteal bone resorptions in lateral aspects of 2nd and 3rd middle phalanges. There were no radiological manifestations of rickets. Metabolic profile revealed hypercalcemia with hypophosphatemia. Intact parathyroid hormone levels were elevated at 790 pg/ml. Vitamin D levels were deficient. She had low bone mineral density with Z score of −3.4. Vitamin D supplementation resulted in worsening of hypercalcemia without reduction in PTH levels. Tc 99 Sestamibi uptake scan showed abnormal tracer retention in left inferior pole of thyroid. A large parathyroid gland was removed with histology favoring parathyroid adenoma. Post operatively she developed hypocalcemia. Bilateral osteotomy was done for SUFE and further surgeries for correction of limb deformities planned.

**Conclusion:**

PHPT in children is usually diagnosed late when irreversible organ damage has occurred. Children can present with non specific symptoms involving gastrointestinal, musculoskeletal, renal and neurological systems. PHPT can cause disarray in bone and epiphysis in children during pubertal growth spurt. Genu valgum and SUFE are rare skeletal manifestations in PHPT and only 10 cases of genu valgum and 9 cases of SUFE have been reported up to now. So far no cases have been reported on epiphyseal displacement of humeri. Awareness regarding the occurrence of these rare skeletal manifestations especially during puberty is important for early diagnosis to prevent irreversible outcomes.

## Background

Primary hyperparathyroidism (PHPT) is a common condition among adults. However it’s an uncommon disorder in children and adolescents associated invariably with delay in diagnosis. This is mainly attributed to incidental detection of hypercalcemia in routine blood investigations done in adults thus allowing diagnosis at an asymptomatic stage. In contrast diagnosis in children is often when they are symptomatic with target organ involvement. It is postulated that puberty may result in unmasking of certain skeletal manifestations which result due to PHPT.

**Table 1:** Metabolic parameters before and after 3 months of vitamin D supplementation

	Before vit D	After vit D
Ionized calcium (1.12–1.32 mmol/l)	1.5	1.7
Calcium/creatinine ratio	0.06	0.29
ALP (182–587 IU/L)	3210	2053
Intact PTH (8–76 pg/ml)	795	892
Vitamin D (<30 nmol/l – deficiency, 30–79 nmol/l – insufficiency, 80–150 nmol/l - sufficiency)	25	110

We present a case of an adolescent with PHPT who presented with rare skeletal manifestations of genu valgum, bilateral SUFE and bilateral epiphyseal displacement of humeri.

## Case presentation

A 12 year old Sri Lankan girl presented with crooked legs and progressive difficulty in walking for 1 year duration. She did not complain of any leg pain, joint pain or proximal muscle weakness. There was no history of trauma, fractures, abdominal pain, vomiting constipation, neck swelling or renal calculi. She had been relatively healthy up to now taking part in athletic events in school until the development of these symptoms. Her menses were regular following attaining menarche at the age of 11 years. She was a product of a non consanguineous marriage and both her siblings were healthy. Family history was nil of note for any renal disease or multiple endocrine neoplasia (MEN). Her diet was deficient in milk and meat products. Bilateral stapling of knees done 6 months back did not lead to any symptomatic resolution. On examination she had bilateral valgus deformities of the knees without any other clinical features of Rickets or dysmorphism. No other joint deformities were noted. Height and weight were age appropriate and she was in Tanner 2 in pubertal development. The rest of the systemic examination was normal.

X ray imaging of the lower limbs revealed bilateral slipped upper femoral epiphysis (SUFE) and bilateral genu valgus deformity of the knees. As SUFE is associated with a wide range of endocrine and metabolic disorders she was screened for these diseases. Ionized calcium levels were 1.5 mmol/l (1–1.3 mmol/l) which was repeated and confirmed. Serum phosphate levels were low at 2.3 mg/dl (2.5–4.5 mg/dl). Serum Alkaline phosphate levels (ALP) were elevated at 3210 U/L (age matched reference range 182–587 U/L). Serum intact Parathyroid Hormone (PTH) levels were 796 pg/ml (8–76 pg/ml) indicating hyperparathyroidism. Calcium creatinine ratio was 0.06 (>0.02 suggestive if primary hyperparathyroidism). Vitamin D levels were 25 nmol/l (<30 nmol/l suggestive of deficiency). Anterior pituitary hormones inclusive of Thyroid function tests, 9 am Cortisol, Follicular Stimulating Hormone, Luteinizing Hormone, Prolactin and baseline hematological indices, serum electrolytes, renal and liver profile were all normal. A skeletal survey revealed evidence of epiphyseal displacement of bilateral humeral heads, rugger jersey spine and subperiosteal bone resorptions involving lateral aspects of 2nd and 3rd phalanges. There were no radiological features of rickets such as widening, cupping or fraying of the metaphysis. An ultrasound scan performed to detect a possible parathyroid adenoma failed to detect any masses. Ultrasound scan of abdomen did not show any evidence of nephrocalcinosis or renal calculi. Bone densitometry of spine revealed low bone mineral density with a Z score of −3.4. At this stage a differential diagnosis of Primary hyperparathyroidism (PHPT) with vitamin D deficiency or Tertiary hyperparathyroidism due to vitamin D deficiency was considered. It was decided to reassess her following adequate supplementation with vitamin D. She was treated with 50,000 IU of vitamin D2 weekly for 8 weeks followed by maintenance therapy of vitamin D3 1000 IU daily Table 1.

**Fig. 1:**
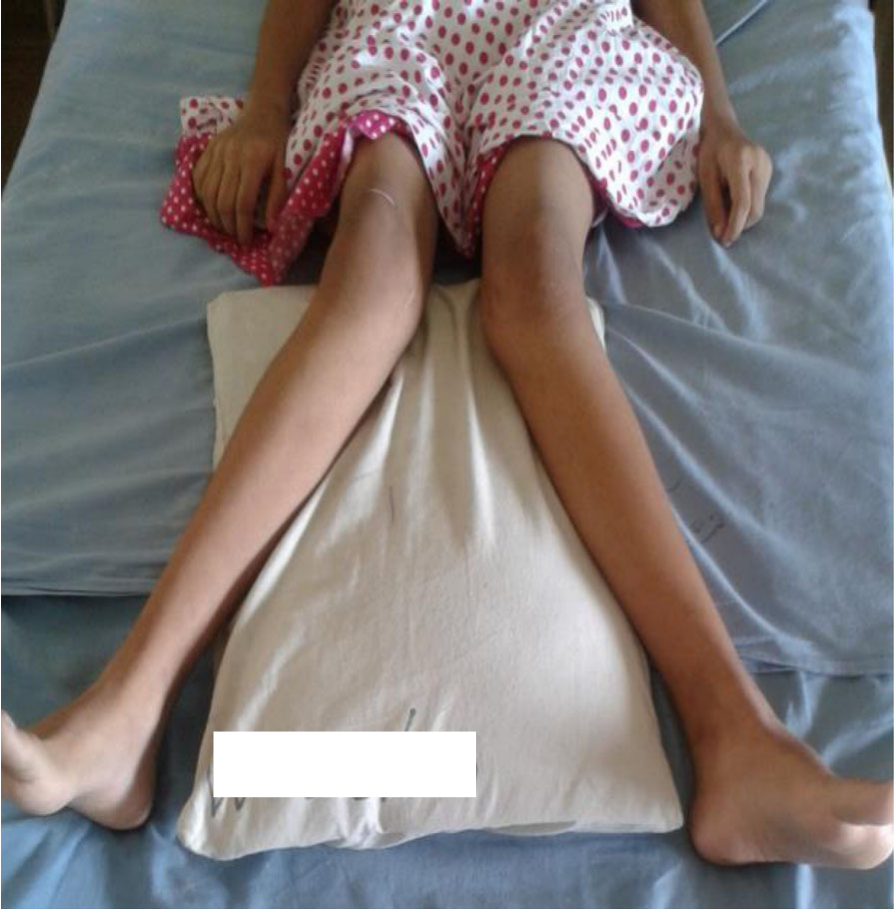
Bilateral genu valgus deformity

**Fig. 2:**
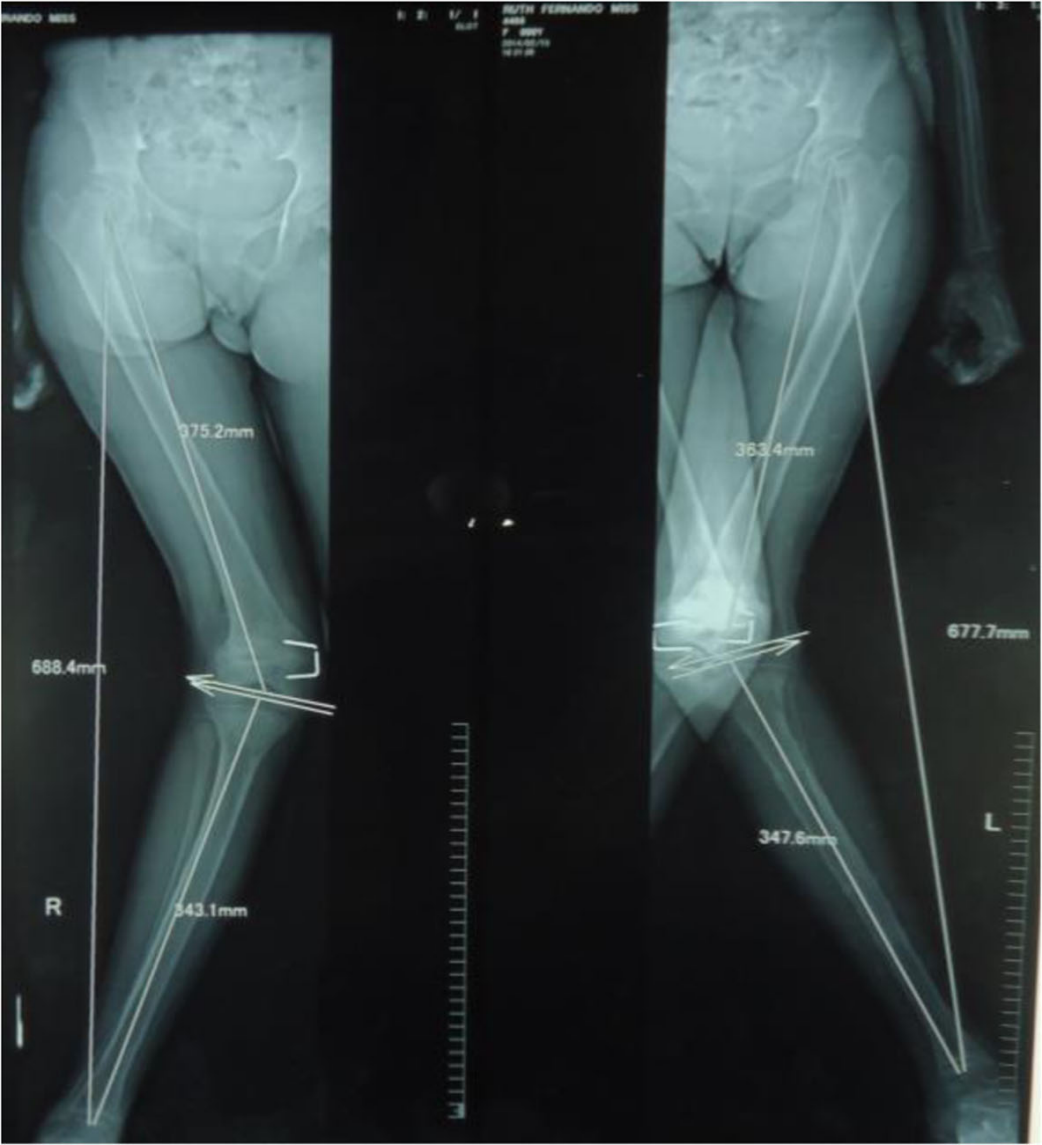
X ray images showing bilateral SUFE and genu valgum

**Fig. 3:**
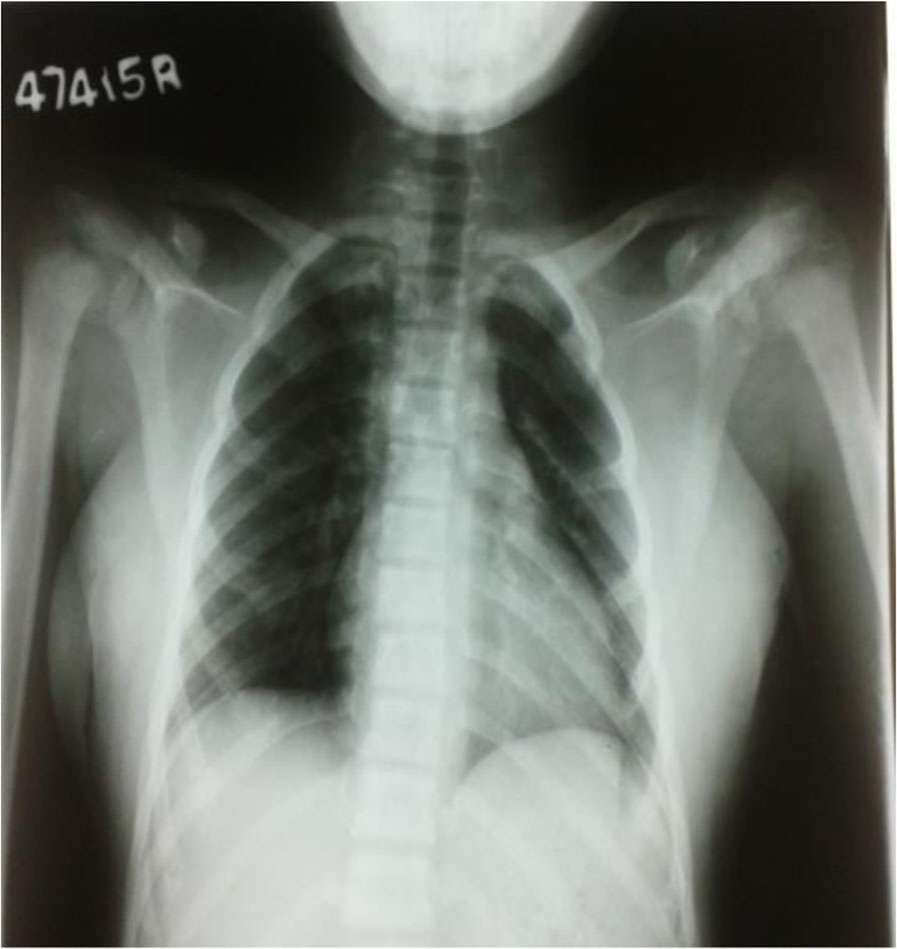
X ray showing epiphyseal displacement of bilateral humeri

**Fig. 4:**
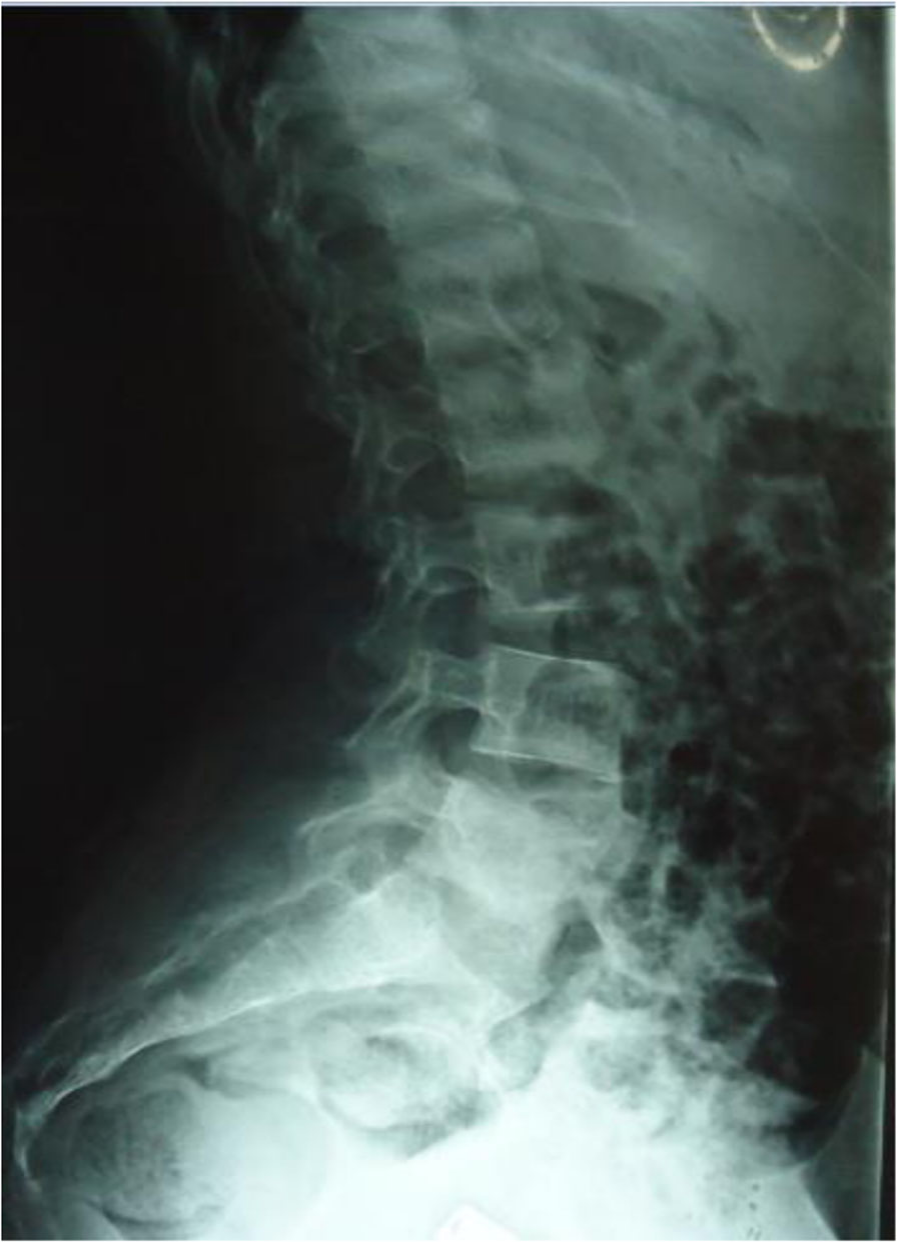
X ray spine showing rugger jersey spine

**Fig. 5:**
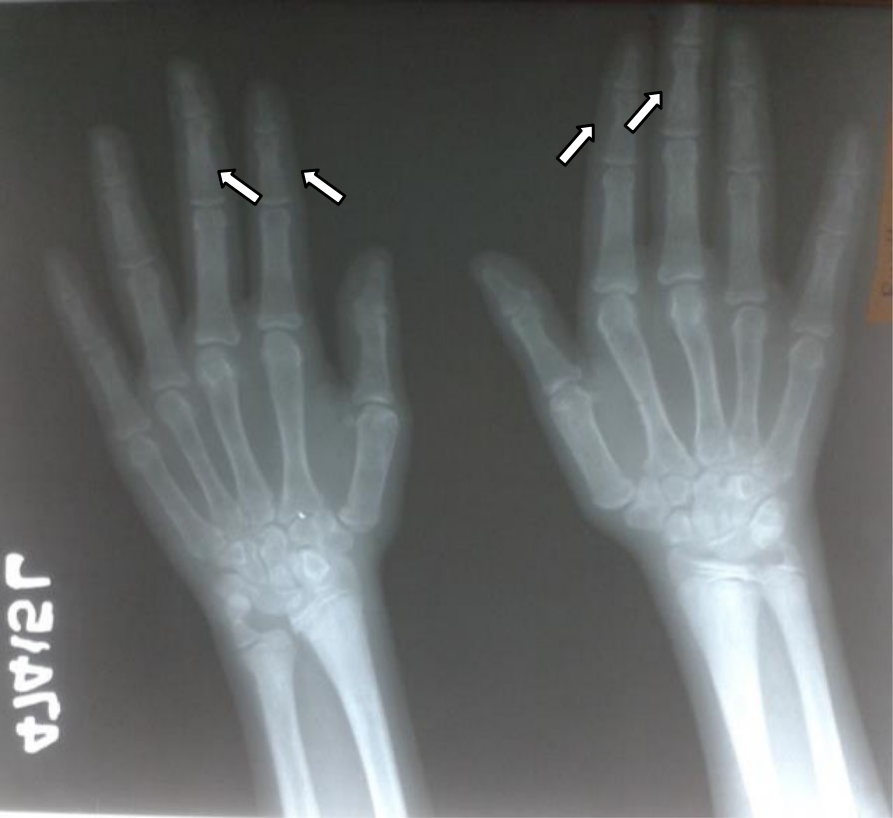
X ray hands showing sub periosteal resorption involving 2nd, 3rd middle phalanges

**Fig. 6:**
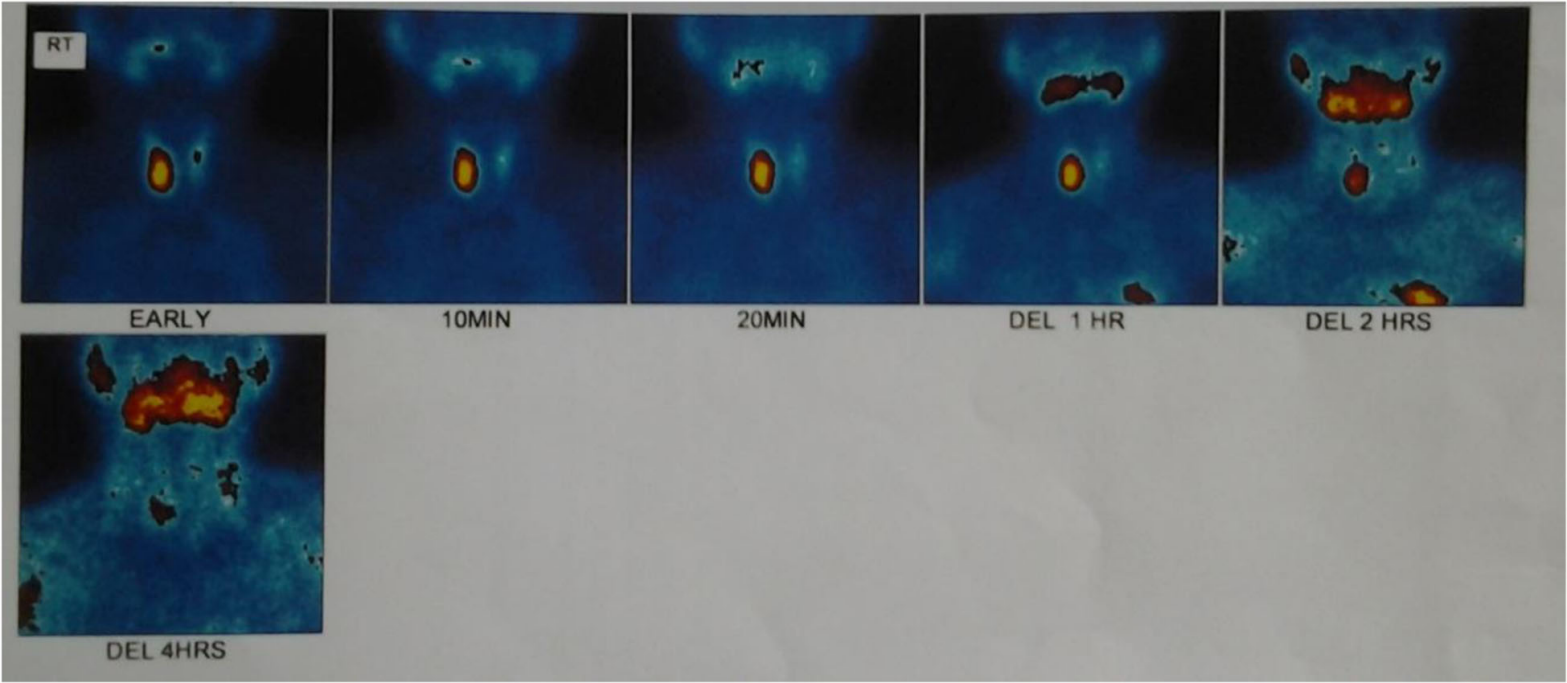
Tc 99 Sestamibi scan showing abnormal, persistent tracer retention in right inferior pole of thyroid

As adequate vitamin D supplementation failed to reduce the PTH levels a diagnosis of primary hyperparathyroidism was made. A Tc99 Sestamibi parathyroid scan revealed focal abnormal and persistent tracer retention in right inferior thyroid pole. As the diagnosis of PHPT was most likely due to parathyroid adenoma she underwent surgery where a large 2 × 3 cm parathyroid gland was removed from the right inferior pole of the thyroid gland. Ipsilateral parathyroid gland was atrophied. Histology favoured a parathyroid adenoma. Post operatively she developed perioral numbness and paresthesia in fingers. Serum ionized calcium levels were 0.8 mmol/l, serum intake PTH levels were 61 pg/ml with normal levels of magnesium 0.82 mmol/l (0.75–0.95 mmol/l) and phosphate 1.0 mmol/l (0.8–1.5 mmol/l). A probable diagnosis of hungry bone disease was made and she was treated with a single dose of intravenous calcium followed by oral calcium carbonate 3 g/day and calcitriol 2 μg/day with monitoring of serum calcium levels. She required calcium supplementation for 3 months following surgery. Bilateral osteotomy was performed for SUFE even prior to completion of metabolic evaluation as delay in treatment can have detrimental effects such as progression of the slippage and avascular necrosis. Series of surgeries for correction of limb deformities were planned following the improvement of quality of bone after treatment of PHPT (Figs. 1, 2, 3, 4, 5 and 6).

## Discussion

PHPT is common disease among adults. However it’s occurrence is very rare among children and adolescents with about 200 cases reported worldwide up to now [1]. It’s incidence is estimated as 1 case per 300,000 live births [2]. PHPT in children has a bimodal age distribution occurring in very young and older children. In neonates and infants it can be due to inactivating mutations of the calcium sensing receptor gene on chromosome 3q which is inherited as an autosomal dominant disorder [3]. In older children it is caused by adenoma or hyperplasia of the gland and can be either familial (27–31%) or sporadic (65–70%).

The characteristic feature in older children is delayed presentation. Almost 80% of children are symptomatic and have end organ damage at the time of presentation [2, 4]. This is in contrast to adults where most of the cases of PHPT are diagnosed by incidental detection of hypercalcemia during routine investigations in asymptomatic patients. Children can present with various non specific symptoms involving gastrointestinal, musculoskeletal, renal and neurological systems due to hypercalcemia. Our patient presented with genu valgum. Skeletal survey revealed bilateral SUFE with bilateral genu valgum, displacement of epiphysis of bilateral humeri, rugger jersey spine and sub periosteal bone resorption involving radial aspects of phalanges in hand. Genu valgum is a rare presentation in children with PHPT with only 10 cases reported in literature. These 10 cases are summarized in an article on Genu valgum and primary hyperparathyroidism in children by Ramkumar et al. [5]. Most of these patients had genu valgum at presentation indicating an etiological link. All these patients had solitary parathyroid adenoma and none were reported vitamin D deficient. Genu valgum can also manifest in vitamin D deficiency which can also cause a similar clinical picture due to tertiary hyperparathyroidism. Our patient had biochemical evidence of vitamin D deficiency. However there were no other radiological features favoring vitamin D deficiency apart from bilateral SUFE which is known to occur in both vitamin D deficiency as well as hyperparathyroidism. Vitamin D deficiency alone is unlikely to cause genu valgum in children with PHPT. Adequate vitamin D supplementation resulted in worsening of hypercalcemia and failure of reduction in PTH levels. Tc 99 Sestamibi uptake scan demonstrated focal increase in uptake of tracer in the right inferior lobe of the thyroid for which she underwent surgery and removal of a parathyroid adenoma with clinical resolution. This was the case in the two patients encountered by Ramkumar et al. as well. The classic picture in the case of tertiary hyperparathyroidism would be parathyroid hyperplasia. Most of the cases of PHPT with genu valgum described so far have occurred in adolescence when a rapid growth spurt is expected. The exact mechanism of genu valgum in PHPT is unclear however it is postulated that PHPT has a direct effect on the growth plate during the pubertal growth spurt [6]. Similar phenomenon occurs in SUFE which is displacement of the capital femoral epiphysis caused by shear stress on a vulnerable physis during rapid growth in adolescence [7]. Although the exact pathogenesis is unclear many endocrine diseases such as hypothyroidism, administration of growth hormone, PHPT, panhypopituitarism and lately vitamin D deficiency have been contributory factors. These disorders result in abnormal growth and mineralization of cartilage thus predisposing to slippage of the vulnerable physis. Prevalences of these among 85 patients with SUFE were hypothyroidism in 40%, growth hormone deficiency in 25% and others in 35% [8]. PHPT causing SUFE is extremely rare with only 9 cases reported worldwide [9]. Madeira et al. reported the occurrence of genu valgum, SUFE and several painful skeletal manifestations in a teenager which was attributed to the rapid growth spurt during puberty [10]. Further to the development of genu valgum and SUFE, our patient had radiological evidence of epiphyseal displacement of bilateral humeri as well. This most likely can be attributed to the same mechanism of the effect of excess parathyroid hormone on the bone and epiphysis in a child going through the pubertal growth spurt. However there is no literature published to date describing epiphyseal displacement of humerus in association with PHPT.

PHPT in our 12 year old patient was unmasked due to these skeletal deformities as she progressed through puberty. Apart from these she did not have any other clinical features suggestive of PHPT. Following excision of the parathyroid adenoma she developed biochemical resolution. However further surgeries will be required to correct the limb deformities.

## Conclusion

PHPT is a rare disease in children which is often diagnosed late once target organ involvement had developed. In contrast, adults with PHPT are incidentally detected in the asymptomatic phase during routine blood investigations. Apart from the well known skeletal manifestations, PHPT can affect the bone and epiphysis resulting in rare skeletal deformities such as genu valgum, SUFE and epiphyseal displacement especially during the pubertal growth spurt in adolescents. Having a high degree of suspicion when children and especially adolescents present with skeletal symptoms may aid in speedy diagnosis and prevention of detrimental long standing deformities.

## Abbreviations

ALP: Alkaline phsophatase
MEN: Multiple endocrine neoplasia
PHPT: Primary hyperparathyroidism
PTH: Parathyroid hormone
SUFE: Subluxation of upper femoral epiphysis
